# RNA Modifications as Drug Targets: Unlocking the Therapeutic Potential of the Epitranscriptome

**DOI:** 10.2174/0113892029406032260414100243

**Published:** 2026-04-17

**Authors:** Dhananjay Taumar, Atul Pratap Singh, Himanchal Sharma, Vatan Chaudhary

**Affiliations:** 1 Department of Pharmacy, IIMT College of Medical Sciences, IIMT University, Meerut, India;; 2 Department of Pharmacy, School of Pharmaceutical Sciences, IIMT University, Meerut, India

**Keywords:** Epitranscriptome, RNA modifications, small molecules, CRISPR, gene expression, therapeutic targets, m6a, disease therapies

## Abstract

**Introduction:**

The epitranscriptome covers reversible changes to RNA. These changes help control gene expression by making RNA more stable, easier to use, or more prone to degradation. They are increasingly implicated in disease development and offer a promising target for treatment. This review covers RNA alterations and their potential clinical applications.

**Methods:**

The systematic analysis of the peer-reviewed literature was performed, including experimental, clinical, and computational research. The biochemical properties and biological functions were used to classify RNA modifications. In addition, we evaluated current therapeutic options, including small molecules, CRISPR/Cas-based technologies, and RNA-targeted approaches.

**Results:**

RNA undergoes immense changes, including N6-methyladenosine (m6A), 5-methylcytosine (m5C), and pseudouridine. By these modifications, the genes are regulated by coordinated writers, erasers, and readers. Targeted therapeutics aim to regulate changes in cancer, neurodegenerative disease, and viral diseases. Several limitations remain, including overly general framing, suboptimal clarity in conveying the message, and inadequate response mechanisms.

**Discussion:**

The epitranscriptome provides an additional regulatory layer with significant therapeutic applications. New techniques appear to be successful. Researchers, in turn, should be more specific. These therapies require improved delivery vehicles and reduced side effects not associated with the treatment to be safe and effective for the patient.

**Conclusion:**

Therapy targeting RNA-based modifications is a breakthrough in disease treatment. These chemical modifications affect gene function and expression patterns. The next step in the research requires overcoming current limitations to fully realize the therapeutic potential of epitranscriptomics-based interventions.

## 1 INTRODUCTION

The epitranscriptome is the set of chemical alterations present in RNA that can influence how each type of RNA is translated into protein, how it functions in a cellular context, and its stability, without altering its underlying genetic code. Epitranscriptomic changes can be categorized into two main categories: chemical modifications and mutations. The main difference between chemical modifications and mutations is that chemical modifications are dynamic and therefore reversible, in contrast to genetic mutations, and can therefore be used to precisely regulate gene expression based on environmental and/or cellular parameters [1, 2]. Over 170 distinct chemical alterations have been recognized across the key classes of RNA, involving mRNA, tRNA, rRNA, and ncRNA. Among the thousands of known RNA modifications, the most-studied include m6A (N6-methyladenosine), m5C (5-methylcytosine), and ψ (pseudouridine), as these adjustments play important roles in regulating gene expression [3]. There are three classes of proteins responsible for adding or removing these RNA modifications: 'writers' (aka methyltransferases), 'erasers' (aka demethylases), and 'readers' (aka RNA-binding proteins). Writers are essentially methyltransferases that add a specific type of modification to RNA (Fig. **1**). Erasers are responsible for removing existing modifications, allowing several variations on a single RNA molecule [4]. Each of these three components can have a profound effect on the stability, localisation, and translation of a given RNA molecule. Ultimately, all three components affect the ultimate fate and functional activity of an RNA molecule and contribute to many important biological activities, including differentiation, metabolic activity, and cellular responses to stress [5].

Numerous disease processes have been linked to epitranscriptomic changes, demonstrating their significance in cellular homeostasis and pathogenesis. In addition to completely disrupting cellular processes, dysregulation of RNA changes may have a role in the development and/or evolution of illnesses such as cancer, neurological disorders, cardiovascular disease, and infectious diseases [6]. Changes in m6A methylation have been linked to both the silencing of tumour suppressor genes and the activation of oncogenes in cancer. For instance, elevated m6A levels in malignancies facilitate the stability of oncogenic transcripts, which in turn promote tumour development and metastasis. However, deletion of m6A may provide resistance to apoptosis, enabling unchecked cell growth [7].

Thus, altering the methylation process by directly targeting the enzymes involved in RNA methylation has been considered a potential therapeutic target in cancer treatment. In addition to oncology, dysregulation of epitranscriptomics has been associated with neurodegenerative diseases like Parkinson's and Alzheimer's. This dysregulation can affect the stress response, synaptic plasticity, and neural development [8]. Furthermore, disturbances in RNA modification patterns have been monitored in the brains of patients with neurodegenerative diseases, suggesting the epitranscriptome may contribute to disease progression. A deeper understanding of how RNA changes affect brain function could open new opportunities to develop treatments for conditions currently considered untreatable. Beyond neurodegeneration, RNA modifications can also affect immune evasion and viral replication during viral infections [9]. For example, viruses like SARS-CoV-2 and HIV might exploit host RNA modification pathways to enhance viral RNA stability and prevent immune clearance. A therapeutic approach to interfere with RNA processing and the ability of viral RNAs to replicate and evade the host immune response may involve targeting epitranscriptomic modifiers [10]. This review focuses on the potential of targeting the epitranscriptome as a novel therapeutic approach, given evidence indicating that RNA alterations are a crucial regulator of disease. Modern medicine has long relied on targeting DNA and proteins, but RNA alterations offer a new layer of gene expression control that may one day be therapeutically modulated. This review will discuss the different types of RNA alterations, their roles in disease, and, from the standpoint of targeting the epitranscriptome, the status of drug development to manipulate RNA modifications through various methods [11]. We will discuss current small-molecule inhibitors of RNA-modifying enzymes, RNA-targeting therapeutics, and emerging technologies, such as CRISPR-based tools, that can more precisely edit RNA modifications through their editing mechanisms. Furthermore, we will also examine challenges in drugging the epitranscriptome, such as specificity, delivery, and off-target effect concerns. Overall, we will explore the potential of RNA modification-based treatments to create new paradigms for conceiving gene expression modification in transforming the treatment of disease and precision medicine [12].

## 2 STUDY DESIGN AND METHODOLOGY

### 2.1 Study Design

This review used a systematic and structured narrative review approach, following the PRISMA framework to ensure transparency, reproducibility, and methodological rigor.

### 2.2 Literature Search Strategy

A thorough and organised literature search was conducted across these databases:

• PubMed/MEDLINE
• Scopus
• The Web of Science
• Google Scholar

The search included studies published up to December 2025.

#### 2.2.1 Search Strategy and Keywords

Boolean operators were used to create search strings:

(“epitranscriptome” OR “RNA changes”)

AND (“m6A” OR “m5C” OR “pseudouridine”)

AND (“RNA methylation” OR “RNA control”)

AND (“therapeutics” OR “drug targeting” OR “CRISPR”)

In addition, we manually reviewed reference lists to identify additional relevant studies.

### 2.3 Inclusion and Exclusion Criteria

#### 2.3.1 Inclusion Criteria

• Peer-reviewed original research and review articles
• Studies investigating alterations to RNA and epitranscriptomics
• Publications addressing therapeutic applications or disease associations
• English-language publications
• Investigations providing experimental, clinical, or computational evidence

#### 2.3.2 Exclusion Criteria

• Editorials, commentaries, and non-peer-reviewed content
• Research unrelated to RNA modifications
• Duplicate records
• Articles lacking sufficient methodological clarity

### 2.4 Study Selection Process

The study selection process was conducted in three stages:

• Identification involved retrieving articles from the chosen databases.
• Screening entailed evaluating titles and abstracts to determine their relevance.
• Eligibility was assessed through a full-text evaluation, adhering to predetermined criteria.
• Inclusion comprised the final selection of studies for qualitative synthesis.

Publications from the last five years and those with high impact were prioritised.

### 2.5 Data Extraction and Synthesis

Data were systematically extracted and categorised based on the type of RNA modification, such as m6A, m5C, and Ψ.

• Enzymatic regulators (readers, writers, and erasers)
• Functions of living things
• Links to diseases
• Strategies for treatment

A thematic synthesis methodology was employed to consolidate findings into organised segments.

### 2.6 Quality Assessment

We looked at the quality of the studies that were included by looking at:

• Indexing of journals (Scopus/Web of Science)
• The robustness of the study design
• Testing in real life
• Importance for medical uses

Recent studies with significant impact had greater leverage.

## 3 RNA MODIFICATIONS AND THEIR FUNCTIONAL ROLES

### 3.1 Types of RNA Modifications

For RNA metabolism and the control of gene expression, RNA modifications are many, varied, and vital. The functional implications of N6-methyladenosine (m6A), 5-methylcytosine (m5C), and pseudo uridine (ψ) in biological processes have drawn the most interest among the many RNA modifications that have been reported [13]. Eukaryotic mRNAs often carry RNA modifications, which are introduced by specific methyltransferase complexes that regulate post-transcriptional gene expression. Using reporter proteins, it has been shown that m6A modification of RNA affects RNA stability, splicing, and translation efficiency. NSUN family methyltransferases add m5C, which is present in mRNA and non-coding RNA and plays crucial roles in RNA translation and stability [14]. A structural isomer of uridine, pseudouridine (ψ), plays a role in ribosome function and tRNA stability by promoting RNA stability and translation efficiency. Other noteworthy RNA modifications, such as N1-methyladenosine (m1A), promote translation initiation, while 2'-O-methylation affects RNA stability and immune recognition. The presence of these RNA modifications means that the functional outcomes of RNA-protein interactions contribute to robust control of gene expression [15].

Beyond the well-characterized m^6^A, m^5^C, and pseudouridine (Ψ), several other RNA mutations have earned attention for their functional significance in health and disease [16]:

• N4-acetylcytidine (ac^4^C): Catalyzed by NAT10, ac^4^C has been shown to increase mRNA stability and translation efficiency. It is enriched in coding regions and influences gene expression under stress and in cancer conditions such as osteosarcoma.
• N1-methyladenosine (m^1^A): Found in tRNA, rRNA, and mRNA, m^1^A plays a role in translation initiation and protein synthesis. Dysregulation has been observed in liver and gastrointestinal cancers.
• 7-methylguanosine (m^7^G): This cap-associated modification is critical for mRNA stability and nuclear export. Internal m^7^G has also been identified in mRNA, influencing translation and gene expression in neurological and oncogenic contexts.
• N6,2′-O-dimethyladenosine (m^6^Am): Positioned adjacent to the m^7^G cap, m^6^Am contributes to transcript stability and has been associated with response to hypoxia and cellular stress. METTL4 and PCIF1 are key enzymes regulating this mark.

### 3.2 Enzymes Involved in RNA Modifications

Epitranscriptomic processes involve three enzyme groups: writers, erasers, and readers. Writers add chemical modifications to RNA [17]; for example, NSUN2 and DNMT2 add m5C, and pseudouridine (Ψ) synthases such as PUS1 convert uridine (U) to Ψ [18]. Erasers are demethylases that remove RNA modifications, rendering epitranscriptomic changes dynamic and reversible. FTO and ALKBH5, for instance, demethylate m6A, a modification linked to RNA metabolism and cellular responses. Readers are RNA-binding proteins that identify specific changes and translate these epitranscriptomic signals into RNA processing and functional outcomes. For example, the YTH domain binds selectively to m6A, regulating RNA stability and translation, while ALYREF binds to m5C and facilitates RNA export [19]. Together, these enzyme groups interact to coordinate modifications that impact RNA fate and function.

### 3.3 Functions in Gene Expression Regulation

These epitranscriptomic changes in RNA, coordinated by writers, erasers, and readers, can have global effects on gene expression. For instance, modifications of cytosine, such as N6-methyladenosine (m6A), lead to RNA degradation mediated by YTHDF proteins that recognize m6A and direct RNAs for degradation, thereby affecting the half-life of a transcript, or how long an RNA molecule remains in the cell. Additionally, other RNA modifications, such as Ψ or 5-methylcytosine, may promote ribosome binding or stabilize RNA secondary structure, thereby enhancing translation. Additionally, m6A and other modifications can affect RNA splicing, the process of removing introns from RNA and joining exons, thereby influencing both conventional and alternative splicing events [20].

### 3.4 Epitranscriptomic Regulation of Cellular Processes

The chemical makeup of RNA has important effects on cellular functions such as differentiation, apoptosis, and responses to the environment. For example, the m6A chemical modification partially regulates embryonic stem cell differentiation by maintaining pluripotency [21]. m6A and other RNA chemical modifications (*e.g*., pseudouridine) help stabilise messenger RNA (mRNA) and enhance translation under cellular stress. This data indicates that an RNA chemical modification can enable the cell to adapt to stress. RNA chemical modifications have significant potential as therapeutic targets for numerous disorders (*e.g*., cancer and neurodegenerative diseases) (Table **1**) [22].

#### 3.4.1 Non-coding RNA Modifications: Emerging Functional Landscapes

While the current literature is largely focused on mRNA, a growing body of research examines the biochemical properties of non-coding RNAs (ncRNAs). There are various types of non-coding RNAs, involving transfer RNAs, ribosomal RNAs, and long non-coding RNAs. Non-coding RNA is not an inert molecule; rather, many ncRNAs play important roles in translation, ribosome assembly, and epigenetic regulation, among others [23].

For example, the chemical modification of the tRNA molecule by NSUN2, which adds 5-methylcytosine (m5C), is required for both stable protein production and cellular resistance to stress. Notably, tRNA m5C alterations have been correlated with a variety of pathological conditions, including cancer and neurodevelopmental disorders. In these cases, dysregulation of tRNA m5C impairs codon recognition and protein synthesis, leading to proteomic imbalance [24].

Ribosomal RNA (rRNA) is a type of RNA involved in making ribosomes, which are cell structures that produce proteins. Two main types of chemical changes, or modifications, occur on rRNA: adding pseudouridine (Ψ, a chemically altered version of the standard RNA building block uridine) and adding methyl groups (small chemical attachments) at specific sites through a process known as 2'-O-methylation. These changes help maintain the correct structure and function of ribosomes. Mutations in enzymes that make these modifications, such as dyskerin and fibrillarin, have been associated with tumorigenesis and ribosomopathies. Studying the patterns of rRNA modification could help diagnose and treat cancer [25].

rRNA modifications, N6 (m6) modifications refer to the adding of a methyl group at the nitrogen-6 position of adenine (one of the four RNA building blocks). When these modifications occur in long non-coding RNAs (lncRNAs), they can change the lncRNA’s three-dimensional shape, how it moves from the cell nucleus to the cytoplasm, and how it interacts with proteins that bind to RNA. As a result, these changes can affect important biological processes, such as inactivating one X chromosome in cells, stem cell development, and processes that drive cancer. For example, an N6-methyladenosine (m6A) alteration in the lncRNA MALAT1 alters its interactions with splicing factors (proteins that control RNA editing), thereby promoting tumor growth. Research into how non-coding RNAs (ncRNAs, RNAs that do not encode proteins) are modified is still limited, but it is important for understanding diseases and developing treatments [26].

### 3.5 Bioinformatics and Computational Approaches in RNA Modification Research

Due to the booming field of epitranscriptomics, bioinformatics, and machine learning applications have played a critical role in identifying, predicting, and analyzing transcriptome-wide RNA modifications. These computational methods were used to complement experimental data, including high-throughput analysis and functional annotation of RNA modification sites across diverse biological and disease settings [34].

#### 3.5.1 Machine Learning and Deep Learning Models

Machine learning and deep learning algorithms now directly improve the accuracy of predicting RNA modification sites. Tools such as DeepRMeth, m6Aboost, and Rm-LR use neural networks, including convolutional neural networks (CNNs) and recurrent neural networks (RNNs), to predict the locations of RNA methylation sites (such as m6A and m5C) based on sequence features, secondary structure, and physicochemical properties. These models improve sensitivity and specificity beyond those of traditional motif-based methods [35].

#### 3.5.2 RNA Modification Prediction Platforms

Several platforms have been created to forecast sites of modification for various RNA species. Training datasets from experimental profiling are used to predict transcriptome-wide maps with sequence-based predictors such as SRAMP (for m6A) and m5UPred (for m5C). It has also been noted that structural predictors have been used to incorporate RNA folding and accessibility characteristics to further enhance the prediction of modification [36].

#### 3.5.3 Databases for RNA Modifications

Public repositories provide curated datasets of experimentally validated and computationally predicted RNA modifications. Notable examples include [37]:

• RMVar: Catalogs RNA modification sites and their functional variations in disease.
• RMBase: Integrates epitranscriptomic data across species and modification types.
• PIANO: Focuses on pseudouridine (Ψ) site prediction and annotation.
• MODOMICS: A comprehensive database of RNA modification pathways, enzymes, and chemical structures.

#### 3.5.4 Functional Annotation Tools

After predicting the sites of RNA modification, iRNA-m6A functional prediction of m6A sites is performed based on the context of 5-methylcytosine sites, and m5C-Atlas provides expression and conservation evidence on the annotated sites. Gene set enrichment tools, such as Enrichr, facilitate the analysis of pathway and gene connectivity between RNA modifications, cellular processes, and disease pathways. Recently developed computational techniques are transforming the study of RNA modification, offering greater resolution of the RNA modification landscape (also known as the epitranscriptome). As datasets expand and algorithms become more sophisticated, bioinformaticians will continue to play a key role in uncovering potential therapeutic targets and developing new therapies targeting RNA modifications [38].

### 3.6 Experimental Techniques for Detecting RNA Modifications

To understand how RNA modifications function biologically and how they relate to disease, accurately identifying them is essential. Several advanced experimental approaches have been developed, each varying in sensitivity, resolution, and specificity. For example, mass spectrometry enables direct and quantitative detection of modifications, RNA sequencing typically provides high-throughput identification of specific modification types, and chemical labeling can be used to selectively enrich or identify modified RNA molecules. These distinctions allow for tailored approaches depending on the scientific question and the type of RNA modification under investigation.

#### 3.6.1 LC-MS/MS (Liquid Chromatography–Tandem Mass Spectrometry)

This gold-standard technique enables direct identification and quantification of RNA modifications at the nucleotide level. After enzymatic digestion of RNA into nucleosides, LC-MS/MS separates and detects modified bases based on mass-to-charge ratios, enabling high specificity and multiparameter detection. However, it cannot pinpoint modification locations and requires complementary approaches, such as RNA sequencing, to map the transcriptome-wide spatial distribution [39].

#### 3.6.2 MeRIP-seq (m6A-seq)

Mapping of N6-methyladenosine (m6A) modifications in the transcriptome can be achieved using methylated RNA immunoprecipitation sequencing (MeRIP-seq). The MeRIP-seq process begins with m6A-specific antibodies that immunoprecipitate m6A-containing RNA, followed by next-generation sequencing of the resulting libraries. This approach yields an enrichment map of m6A across the transcriptome. However, limitations include relatively low resolution (100 nt), which limits precise localisation, and antibody-specific issues, such as cross-reactivity and off-target binding, which complicate data interpretation and reduce site-level confidence. Nevertheless, MeRIP-seq provides valuable insight into dynamic m6A changes under biological and disease conditions [40].

#### 3.6.3 Nanopore Direct RNA Sequencing

Nanopore sequencing offers a real-time, amplification-free solution for detecting RNA modifications in native RNA. In contrast, traditional methods require reverse transcription or chemical modification, which introduces biases and eliminates direct information about the original nucleotides. Nanopore sequencing directly reads unmodified RNA as it passes through a protein nanopore. This enables detection of base modifications (*e.g*., m6A, Psi, m5C) *via* changes in electrical current. Although it achieves single-molecule resolution and preserves modification status, distinguishing modification types remains challenging due to overlapping signals and noise. Basecalling algorithms may also introduce errors, affecting modification detection. Nonetheless, nanopore sequencing remains a valuable tool for studying modification dynamics specific to individual transcripts [41].

Complementary technologies have revolutionised epitranscriptomics by enabling high-resolution, modification-specific, transcriptome-wide analysis. Combining these with computational modelling and machine learning allows systematic annotation and functional inference of RNA modifications across various physiological and pathological conditions.

## 4 RNA MODIFICATIONS IN DISEASE PATHOGENESIS

### 4.1 Cancer

RNA alterations influence cellular signalling, RNA stability, and gene expression, all of which are crucial for the pathophysiology of cancer. N6-methyladenosine (m6A) is the most researched RNA alteration in cancer, even though there are many others [42]. Three different protein types are involved in the dynamic control of m6A: molecular writers (methyltransferases, such as METTL3 and METTL14), molecular erasers (demethylases, like FTO and ALKBH5), and molecular readers (RNA-binding proteins, including YTHDF1 and YTHDF2). Alterations in m6A methylation can silence tumour suppressors or activate oncogenes in various malignancies, resulting from abnormalities in writer, eraser, and reader proteins [43]. For example, the writer protein METTL3 is upregulated in acute myeloid leukaemia (AML), enhancing leukemic cell proliferation by stabilising carcinogenic transcripts [44]. Similarly, the eraser FTO increases the expression of carcinogenic genes by removing m6A modifications and promotes tumour growth in breast cancer and glioblastoma.

In contrast, m6A modifications can sometimes act as tumour suppressors by promoting the degradation of carcinogenic mRNAs through YTHDF2 and related pathways, despite m6A being generally associated with oncogenesis [45]. Finally, other RNA modifications linked to cancer include pseudouridine (Ψ) and 5-methylcytosine (m5C). NSUN2, a well-known m5C writer, stabilises transcripts implicated in cellular proliferation and is overexpressed in bladder and colorectal malignancies. Changes in pseudouridine synthases may also affect RNA stability or translation efficiency, leading to tumour development [46]. Small-molecule inhibitors of METTL3 and FTO are the subject of active preclinical research, and interventions targeting RNA alterations may constitute the next frontier of cancer treatments. Lastly, a novel technical strategy for altering RNA methylation patterns and maybe reestablishing normal gene expression in cancer cells is CRISPR-based epitranscriptomic editing (Fig. **2**) [47]. It is critical to understand that, depending on the cellular milieu, tissue type, and stage of malignancy, A may behave as a tumour suppressor or an oncogenic driver in cancer biology. For instance, METTL3, a crucial enzyme in acute myeloid leukaemia (AML), promotes leukemogenesis and stem cell proliferation by enhancing translation of oncogenes such as MYC and BCL2 [48].

Silencing METTL3 in AML blocks leukemic progression, demonstrating its oncogenic driver status. In glioblastoma, the m6A reader protein YTHDF2 promotes the degradation of pro-oncogenic transcripts, thereby suppressing tumour growth. When YTHDF2 is knocked out in glioma models, transcripts stabilise, and tumours grow, leading to resistance [49]. Similarly, changes in m6A-related enzymes directly affect cell survival, apoptosis, and metastasis in hepatocellular carcinoma and colorectal cancer, underscoring the highly context-specific nature of m6A biology. These opposing roles show the need for tumour-type-specific targeting and precise diagnostics to determine when mOA modulation is helpful or harmful. Detailed profiling of the epitranscriptomic environment, including m6A writers, erasers, and readers in each cancer subtype, should guide therapeutic interventions.

### 4.2 Neurodegenerative Diseases

Much research is beginning to show that neurodegenerative diseases such as Alzheimer’s Disease (AD), Parkinson’s Disease (PD), and Amyotrophic Lateral Sclerosis (ALS) can be affected by RNA modifications [50]. Because the brain relies heavily on RNA processing, even minor changes in this process can cause profound alterations in neuronal activity. One prominent example is m6A, a well-studied RNA modification associated with neurodegenerative diseases. Specifically, m6A has been shown to regulate synaptic plasticity, memory, and neuronal survival [51]. Focusing on AD, evidence indicates that dysregulated m6A impairs the clearance of amyloid-beta (Aβ) plaques from the brain and promotes the aggregation of tau proteins into neurofibrillary tangles, both of which are believed to be hallmarks of AD. Similarly, loss of m6A methylation in PD has been linked to misfolding of alpha-synuclein, a core protein implicated in the progression of PD [52]. Pseudo uridine (Ψ) is also a well-studied RNA modification that is important for the stability and functionality of neuronal RNA. Like m6A, pseudo uridine modification has been shown to mediate RNA-binding proteins, such as TDP-43 and FUS, in ALS, where abnormalities in pseudo modification lead to mislocalised or dysfunctional RNA modifications [53]. The resulting RNA instability leads to progressive motor neuron degeneration. The instability of the resulting RNA ultimately results in progressive degeneration of motor neurons. In terms of neuroprotective therapy, focusing on epitranscriptomic pathways offers a promising approach. Investigational small molecules that modulate RNA methylation are being evaluated as potential means to restore normal RNA processing in neurons. Furthermore, RNA-based therapeutics are an alternative approach using antisense oligonucleotides (ASOs), which are currently being studied as therapeutic candidates to correct RNA modifications in neurodegenerative conditions [54].

In the context of viral infections, RNA viruses depend on host RNA modifications to replicate and evade host immune recognition. Several viruses, including HIV, SARS-CoV-2, and influenza, utilize the host’s epitranscriptomic pathway to stabilize viral RNA and enhance translation of the viral RNA [55]. One of the main RNA modifications found to play a role during viral infection is m6A, which can modulate viral RNA processing and enhance immune detection. For example, HIV-1 RNA contains m6A marks that enhance nuclear export and translation, leading to increased viral replication. The coronavirus SARS-CoV-2 (or COVID-19) exploits m6A modifications to stabilize the virally encoded RNA and enhance infection efficiency [56]. Notably, while some viruses utilize m6A modifications, other viruses inhibit this signal. For example, the influenza A virus inhibits m6A methylation in the host, thereby evading immune detection. This illustrates the dual role of RNA modifications during viral infection, as they can support viral replication or, depending on the virus, tissues, or cell type, even inhibit it [57]. Overall, RNA modifications can be a good target for antiviral therapeutics. Preclinical data have shown that small-molecule inhibitors of m6A-modifying enzymes (*e.g*., FTO inhibitors) reduce viral replication *in vitro,* and RNA editing technologies (*e.g*., CRISPR-based methods) also offer a pathway for precise control of viral RNA modification, thereby providing new directions for antiviral therapeutics [58].

### 4.3 Cardiovascular Diseases

Recent studies have shown that RNA modifications are relevant in cardiovascular disease (CVD), such as heart failure, atherosclerosis, and hypertension. The modifications m6A, m5C, and pseudo-uridine regulate essential cardiac-to-vascular functional transcripts and those involved in inflammation [59]. In heart disease, m6A modifications may be modulating gene expression in cardiac myocytes and endothelial cells. In addition, evidence suggests that alterations to m6A methylation may serve as a marking for cardiac hypertrophy or heart failure and ischemic injury [60]. For example, increased m6A levels were found in cardiac cells, leading to altered expression of genes related to fibrosis and hypertrophic remodelling, thereby accelerating heart disease onset and progression [61]. In atherosclerosis, which is a major cause of cardiovascular disease, vascular inflammation and plaque formation may be due to abnormal RNA modifications. The RNA modification m5C was shown to regulate inflammation in endothelial cells, with higher m5C levels positively correlated with inflammatory cytokines. Pseudo uridine may also contribute to vascular function and cholesterol metabolism. Enzymes that may modify pseudouridine have been implicated in endothelial dysfunction and vascular susceptibility [62]. Given the role of RNA modifications in heart disease, efforts are underway to target therapeutic interventions to epitranscriptomic pathways. Investigational small-molecule inhibitors and RNA-targeting therapies are underway to potentially normalize aberrant RNA modifications and quality. In addition, RNA-based therapeutics, such as mRNA vaccines or RNA-targeting agents, represent a novel and exciting method for treating heart disease at the molecular level (Table **2**) [63].

## 5 TARGETING THE EPITRANSCRIPTOME

### 5.1 Challenges in Targeting RNA Modifications

Utilizing RNA modifications for therapeutic applications comes with considerable hurdles. In contrast to DNA modifications, which are more stable and have been well-studied, RNA modifications are dynamic, reversible, and cell-type specific, all of which complicate the development of drugs that can target RNA modifications specifically and without off-target effects [76]. One of the most significant challenges to overcome is specificity. Many RNA modifications occur at multiple sites across all classes of RNA (mRNA, rRNA, tRNA, and non-coding RNAs), and a single chemical modification can have differential downstream effects depending on the cellular context [77]. For example, m6A modifications in mRNA affect the expression of the gene, while m6A in long non-coding RNA (lncRNA) changes RNA-protein interactions. Developing drugs that can inhibit or enhance a particular modification specifically and without impacting other important cell functions is a major challenge. Another challenge is drug delivery [78]. Unlike current DNA-targeting therapies that utilize nuclear localization signals for delivery into the cell nucleus, RNA-modifying or targeting enzymes can act in different cellular spaces, including the nucleus, the cytoplasm, and the mitochondria. Introducing small molecules or RNA-based therapeutics into these compartments with great efficiency is very challenging. Work is currently being done using lipid carriers and/or nanoparticle-based delivery systems to improve the stability and targeted delivery of RNA-modifying therapeutics [79]. Furthermore, RNA modifications are often transient, dynamic, and regulated, meaning that a drug must be sensitive to dosing to avoid over-inhibition or inhibiting for an excessive amount of time. This is relevant for m6A modifications, where either excessive or inadequate methylation can lead to unfavourable outcomes. Drug specificity and toxicity remain important concerns as unintended modifications of non-target RNAs may alter certain cellular processes, causing side effects [80]. Even while this remains a challenge, computational modelling, RNA structure prediction, and high-throughput screening are emergent tools that may help break down some of the barriers discussed [81]. As drug design improves, it is anticipated that drugs will be developed that inhibit RNA-modifying enzymes more specifically and more precisely, which may result in future therapeutic avenues [82].

#### 5.1.1 Combination Therapy Strategies in RNA Modification-Based Therapies

A more promising approach to circumvent resistance and improve therapeutic response in cancer and other diseases is combination therapy using RNA-modifying enzyme inhibitors alongside other treatment modalities. Some of the promising combination therapies that have been reported in preclinical and clinical trials are listed below:

##### 5.1.1.1 METTL3 Inhibitors + Radiotherapy in Solid Tumours

METTL3, a major enzyme involved in m6A methylation (the addition of a methyl group to messenger RNA), has been shown to control the expression of oncogenic (cancer-causing) genes and tumour development in many cancers. In solid tumours, such as non-small cell lung cancer (NSCLC), a common type of lung cancer, METTL3 inhibitors, such as STM2457 (a compound that blocks this enzyme), are used in combination with radiotherapy. This combination increases radiation sensitivity by altering the expression of tumour suppressor genes and limiting DNA repair. Thus, the idea behind this combination therapy is to overcome the resistance often seen with single-agent treatments [83].

##### 5.1.1.2 FTO Inhibitors + Immune Checkpoint Blockade (e.g., anti-PD-1)

Another m6A demethylase associated with tumour progression and immunogenicity is FTO (Fat Mass and Obesity-associated protein). It has been demonstrated that R-2HG, as an FTO inhibitor, can restore m6A levels on gene firewalling immune-related genes, thereby modulating immune responses. This type of combination therapy, combined with immune checkpoint inhibitors (*e.g*., anti-PD-1), can be used to reinvigorate T-cell-mediated anti-tumour immunity by evading tumour immune defence mechanisms [84].

### 5.2 Small Molecule Inhibitors and Activators

Small-molecule inhibitors and activators in biology are designed to target certain small molecules and change how proteins or other molecules interact with their environment. Significant progress has been made in developing small molecules that target the writers (methyltransferases), erasers (demethylases), and readers (RNA-binding proteins) involved in RNA modifications [85]. These molecules either block or promote specific alterations, which in turn affect expression and cellular activity [86].

METTL3, the main enzyme that adds m6A marks to RNA, is one of the most extensively studied targets [87]. A selective METTL3 inhibitor, STM2457, is effective in preclinical studies of acute myeloid leukaemia (AML) by inhibiting oncogenic mRNA translation [88]. Likewise, inhibiting the m6A demethylase FTO has been shown to suppress tumour growth by stabilising tumour suppressor mRNAs. Two FTO inhibitors, FB23 and R-2HG, are currently under study as anticancer agents [89]. In addition to m6A, other RNA modifications are also targeted with inhibitors. For example, NSUN2 inhibitors, which prevent m5C methylation, are under investigation for anti-cancer effects [90]. Similarly, pseudouridine synthase inhibitors are considered potential antiviral agents because viruses often hijack host pseudouridine modifications to aid their replication [91]. While small-molecule inhibitors have been used effectively, activators of RNA modifications are also being explored. For example, enhancing m6A methylation is a potential treatment for neurodegenerative diseases in which RNA remodelling is impaired [92]. Identifying small molecules that increase METTL3 activity could help restore normal RNA processing in disorders such as Alzheimer’s disease and ALS. Although these advances have been made, ensuring the specificity of these inhibitors and activators remains a challenge. The structural similarities between RNA-modifying enzymes and other methyltransferases or demethylases increase the risk of off-target effects. Greater optimisation and structure-based drug design are needed to improve selectivity and therapeutic potential [93].

#### 5.2.1 Emerging PROTAC Technologies: Targeting RNA-Binding Proteins

PROTACs are a novel class of therapeutic molecules that can selectively degrade RNA-binding proteins (RBPs) *via* the ubiquitin-proteasome degradation pathway. The general structure of PROTACs consists of three functional groups: a ligand that interacts with the target protein, a ligand that interacts with an E3 ubiquitin ligase, and a linker that connects the two. The PROTAC forms a tertiary complex between the target protein and the E3 ligase, enabling ubiquitination and subsequent proteasomal degradation of the target [94].

The degradation of RNA-modifying proteins, especially m6A readers such as YTHDF1, is a promising application of PROTACs. YTHDF1 is involved in the regulation of m6A recognition and translation, which in turn modulates multiple biological functions, including tumour growth and metastasis. The inhibition of YTHDF1 with RNA-PROTACs could be a therapeutic approach for cancers in which gene expression regulated by m6A is altered [95]. YTHDF1-targeting RNA-PROTACs have been designed in most cases and consist of chimeric molecules that specifically bind YTHDF1 and recruit an E3 ligase, such as CRBN (cereblon), which mediates ubiquitin-mediated degradation. These RNA-PROTACs can specifically degrade YTHDF1, thereby interfering with m6A-mediated translation control and inhibiting tumour activities of cancer cells [96]. YTHDF1-targeting PROTACs have shown promising preclinical results in breast cancer models, degrading YTHDF1, reducing oncogenic mRNA stability, and inhibiting cancer cell proliferation. RNA-PROTACs have been combined with current therapies, including chemotherapy and immune checkpoint inhibitors, to enhance treatment efficacy and overcome resistance mechanisms [97].

### 5.3 CRISPR/Cas9 and RNA Modification Tools

The invention of CRISPR-based technologies has transformed the analysis and editing of RNA alterations. Although CRISPR/Cas9 is most commonly recognised for its DNA editing functionality, more recent variants of the technology, such as CRISPR-dCas13 (which is catalytically inactive), can precisely target and edit RNA on a genome-wide scale without altering the genome [98].

CRISPR-based systems can be designed to insert or delete specific RNA epimerisations by fusing inactive Cas proteins to RNA-modifying enzymes. An example is when the CRISPR-dCas13 complex, in combination with METTL3, can induce m6A marks at specific sites in RNA, allowing scientists to investigate their effects. Researchers may conveniently erase m6A modifications using CRISPR-dCas13 tethered to FTO to regulate gene activity at any location [99]. In addition to CRISPR, information on RNA-targeting base editors (ADAR-based systems), which induce site-specific nucleotide titles on RNA strands, has been obtained. Combining these technologies enables fixing disease-causing mutations in the genome in a site-specific manner, without permanently altering it, potentially curing diseases where temporary adjustments would be desirable. Although CRISPR-based RNA editing is an exciting RNA-targeting technology, delivery efficiency, immune responses, and off-target effects remain major obstacles. Advancements in the design of guide RNAs and delivery methods, alongside enzymatic engineering, will, over time, eliminate obstacles to therapeutic applications in these fields [100].

### 5.4 RNA Modification-Specific Therapeutics

The development of therapeutics that directly modulate RNA modifications represents a promising and emerging area of research. A variety of strategies are being considered, such as small RNAs, engineered proteins, and antisense oligonucleotides (ASOs) [101]. ASOs may be the most promising strategy. ASOs are short, synthetic RNA or DNA molecules that alter function by targeting RNA sequences. ASOs can be utilised to inhibit RNA-binding proteins that can recognise modified RNAs, thereby altering RNA stability and translation. The most well-established ASOs are FDA-approved therapies for spinal muscular atrophy (Spinraza) and Duchenne muscular dystrophy (Eteplirsen), opening the door for further RNA-targeting drug development [102]. Another strategy is to use RNA-binding proteins engineered to recognise and modify specific RNA sites. These engineered proteins can be designed to selectively add or remove methylation marks, providing a mechanism for specific epitranscriptome regulation [103]. For instance, synthetic m6A reader proteins fused to degradation domains could be deployed to selectively degrade disease-associated modified RNAs. Small interfering RNAs (siRNAs) and microRNAs (miRNAs) can also be used to target RNA and to regulate expression of RNA-modifying enzymes [104]. For example, siRNA could be employed to selectively silence overactive methyltransferases or demethylases, leading to restoration of normal RNA modification patterns in the context of cancer or neurodegeneration. While these advances have been made, challenges remain concerning the efficient delivery of RNAs, off-target effects, and sustained therapeutic effects [105]. Nevertheless, rapid advances in RNA nanotechnology, lipid nanoparticle (LNP) delivery systems, and modified RNA therapeutics present exciting possibilities to translate these strategies into clinical use.

## 6 CASE STUDIES OF EPITRANSCRIPTOME DRUG DISCOVERY

### 6.1 Targeting m6A Modification in Cancer

The identification that N6-methyladenosine (m6A) is important in cancer progression has driven a great deal of drug development targeting RNA methylation pathways. m6A marks can govern mRNA stability, translation, and degradation, therefore playing a central role in the survival and proliferation of cancer cells. Many cancer types, including acute myeloid leukemia (AML), glioblastoma, lung cancer, and breast cancer, show dysregulated m6A machinery, making them rich targets for therapy [106].

One of the most notable drug candidates is STM2457, a selective small-molecule inhibitor of METTL3, the primary m6A writer enzyme. METTL3 is often overexpressed in AML, where it promotes the stabilisation of oncogenic transcripts that drive leukemia cell proliferation. In preclinical studies, STM2457 significantly reduced leukemic cell growth, impaired the self-renewal of leukemia stem cells, and increased survival rates in AML mouse models [107]. The success of STM2457 demonstrates the potential of targeting m6A methylation as a novel cancer therapy. Another target is FTO, an m6A eraser enzyme that is overactive in multiple cancers, including glioblastoma and breast cancer. R-2-hydroxyglutarate (R-2HG) has been identified as an indirect inhibitor of FTO, leading to increased m6A levels in oncogenic transcripts, which promotes their degradation and reduces tumour growth [108]. In glioblastoma models, treatment with R-2HG was shown to inhibit tumour progression by stabilising tumour suppressive mRNAs, suggesting R-2HG has potential for translation into the clinic. In addition to small molecule inhibitors, CRISPR-based epitranscriptomic editing techniques are being investigated for altering m6A levels in cancer cells [109]. For example, CRISPR/Cas13 coupled to METTL3 or FTO can exert artificial control over m6A methylation at specific mRNA sites, allowing precise gene regulation for cancer therapeutic applications. While there has been significant promise targeting m6A modifications, several important hurdles remain, such as ensuring specificity, avoiding off-target effects, and building effective delivery systems for drugs. Despite these challenges, future ongoing clinical studies indicate RNA modification-based therapies could be the future of innovative cancer therapeutics in the future years [110].

### 6.2 Therapeutics for Neurodegenerative Diseases

The role of RNA modifications extends to their involvement in neurodegenerative diseases such as AD, PD, and ALS. The hallmarks of these diseases include progressive neuronal degeneration, impaired RNA processing, and dysregulated protein synthesis, all of which are mediated by the epitranscriptome [111]. One of the more compelling studies on AD indicated that m6A modifications regulate the aggregation of the Aβ and tau proteins that contribute to AD pathology. In this study, altered m6A levels led to impaired clearance of toxic Aβ peptides, exacerbating their toxicity [112]. To remedy the toxicity caused by m6A dysregulation, scientists are developing small-molecule activators of METTL3 to restore m6A methylation of transcripts in neurons and improve protein clearance pathways [113]. In PD, studies have found that m6A dysregulation contributes to the misfolding of alpha-synuclein, leading to the formation of toxic aggregates in neurons. Scientists are also exploring small molecules that modulate the m6A pathways that could reduce the neurotoxicity associated with alpha-synuclein, leading to enhanced function of the dopamine neuron. Indeed, m6A-enhancing small molecules have improved motor function and neuroprotection in various experimental models of PD and have potential therapeutic value in targeting reversible RNA modifications in a disease model, such as PD [114]. Another nascent area is the exploration of antipode oligonucleotides (ASOs) and RNA-targeting drugs to ameliorate RNA modifications related to ALS. In ∆ALS, RNA-binding proteins, such as TDP-43 and FUS, deregulate their native function, resulting in a global instance of RNA instability. Biochemical therapeutics based on ASOs that precisely target modification types associated with these proteins have been revealed as an innovative way to stabilise neuronal RNA transcripts and mitigate disease [115]. Given that epitranscriptomic therapeutics for neurodegeneration are in the early stages of research, there is guarded optimism, supported by the recent success of mRNA-based vaccines and RNA-targeting drugs in other conditions [116]. Future research will be focused on improving the delivery of these drugs to the brain, converging efforts to minimise off-target effects, and optimising RNA modification targeting compounds for use within the clinic.

### 6.3 Viral Infections and Epitranscriptome

RNA viruses, including HIV, influenza, and SARS-CoV-2 (COVID-19), are known to use host RNA modifications to facilitate their replication and immune evasion mechanisms. Consequently, researchers have begun to explore targeting the epitranscriptome as an antiviral approach [117]. One of the most well-researched RNA modifications in the context of viral infections is m6A, which serves an essential function in viral RNA stability, immune recognition, and replication efficiency. For example, studies on HIV-1 have demonstrated that m6A marks on viral RNA promote translation and stability, facilitating productive viral replication [118]. An m6A-targeted epitranscriptomic approach to antiviral therapy has potential and may offer a method to slow the replication of HIV without interfering with host cellular pathways. A key finding in COVID-19 research was the demonstration that SARS-CoV-2 RNA is heavily marked with m6A marks, which help stabilise viral transcripts and facilitate replication. Indeed, studies have shown that inhibiting m6A reader proteins, such as YTHDF1, greatly reduces viral replication in infected cells. It is thus possible that the m6A RNA modification pathway will lead to potential inhibitors of m6A that could be developed as antiviral agents in the future [119].

In addition to m6A, other RNA modifications, including pseudo-uridine (Ψ), also contribute to a virus's evasion of immune detection efforts. Pseudo uridine modification of viral RNA contributes to immune evasion *via* decreasing detection by immune receptors, increasing stability, and therefore increasing infection time [120]. Recently, the ability to target pseudo uridine synthases has been studied as a potential therapeutics through the disassembly of these processes, and during this disassembly, improved immune detection and elimination of viruses can be activated. However, often overlooked, mRNA vaccines have used RNA modification to improve vaccine efficacy. N1-methylpseudouridine, an improved mRNA, is used in the Pfizer-BioNTech and Moderna COVID-19 vaccines to improve stability and translation effectiveness while removing immune detection activation [121]. This clearly demonstrates that charged RNA modification-based therapies utilized to improve immune clearance of viral infections and RNA modification can enhance the performance of vaccines by increasing stability, not immune detection activation, and translation efficiency [122]. While the research realm of epitranscriptomic-based therapeutic antiviral therapies continues to grow and expand, scientists are also working on optimising entry of modified therapies in terms of frequency of dosing and route of administration. Scientists are ramping up the development of small molecules, CRISPR-based RNA modification tools, and siRNA therapies to selectively target viral RNA modifications. Drug specificity and predictability, potential resistance to modifications, and different routes of drug delivery all stand as challenges to the response of these novel therapeutic strategies to fully understand how targeted RNA modification can affect a potentially wide range of variability to target infections. The capability of targeted RNA modifications to affect the outcome of viral infections can ultimately enable new capabilities to treat an array of viral infections [123].

## 7 CURRENT AND FUTURE CHALLENGES

It remains a tremendous hurdle to develop RNA-modifying therapeutics that use targeting with precision and selectivity in terms of effect and fate. RNA modifications such as m6A, m5C, and pseudo uridine are dynamic modifications on multiple RNA species and are often regulated in a tissue-specific manner. The same modification can have different functional outcomes based on the cell type, thus creating a significant barrier to the development of effective drugs that only target the desired modification without impacting additional modifications within the same or other tissue types. Specificity is vital to ensure RNA-modifying therapeutics do not inadvertently modify RNA molecules that are not targeted, which will disrupt critical cellular processes, ultimately leading to significant effects unrelated to the target RNA modification. This challenge will require the development of extraordinarily specific inhibitors or activators that target only the RNA-modifying enzymes or reader proteins associated with a disease process that do not impact the rest of the cell processes and do not produce other unwanted side effects. Another challenge is the delivery methods of the RNA-targeting drug compounds. Many therapeutic compounds are RNA-based and require targeted delivery to specific tissues or cell types to achieve their desired effects. This is particularly challenging for RNA-modifying drug approaches, which often face limitations such as large molecular size, instability, and the potential to trigger immune responses. Additionally, challenges such as tissue-specific variability in RNA modifications, limitations in current gene-editing technologies, and efficient drug delivery remain significant barriers to clinical translation. Unlike small-molecule drug treatment, RNA-targeting therapeutics, comprising small RNAs, antisense oligonucleotides, and CRISPR-based systems, will all need to have an efficient, targeted, and effective delivery method. Nanoparticle carriers, lipid nanoparticles (LNPs), and viral vectors represent some of the alternative methods under study, but these can still face challenges related to penetration of tissue, circulation stability, and immunogenicity. Additionally, as with any targeted therapy, there is the possibility of resistance or adaptation. Cells and viruses are highly efficient at adapting to their environment, including an adaptation to alterations in RNA editing pathways. Occasionally, cancer cells may modify or mutate the expression of RNA-modifying enzymes to escape therapy and generate resistance over time. Similarly, viruses may develop approaches to circumvent the inhibition of RNA modification pathways required for viral replication. To address resistance, we need to closely examine its mechanisms and develop a combination therapy that either prevents or overcomes it. As therapeutics targeting RNA changes move towards clinical use, the major challenge is the potential for resistance, a phenomenon well documented in other precision medicine approaches. Mutations in RNA-modifying enzymes that alter drug-binding sites or enzymatic activity can lead to resistance. For example, mutations in METTL3 structure may reduce the effectiveness of METTL3 inhibitors by altering the binding specificity or the catalytic active site, thereby allowing methylation to persist despite treatment.

The second approach is to activate compensatory or redundant processes. FTO inhibitor-treated glioblastoma cells have been shown to acquire an adaptive up-regulation of ALKBH5, another m6. A demethylase: this up-regulation replenishes the activity of demethylation and stabilises oncogenic transcripts. This plasticity of the epitranscriptomic network also highlights its complexity, in which the repression of one element can be overcome by upregulating co-expressed enzymes or RNA-binding proteins. Moreover, resistance can arise from epigenetic reprogramming, in which cancer cells shift their reliance from one axis of RNA modifications to the other, and vice versa, thereby avoiding the silenced pathway. Inhibition of m6A has been shown to stimulate other mRNA-stabilisation processes in some tumour models, leading to decreased treatment response.

These resistance cases highlight the importance of biomarker-guided treatment selection and combination regimens. Resistance risk may be reduced by blocking multiple pathways or by combining RNA-modifying inhibitors with therapies such as chemotherapy, radiation, or immune checkpoint blockade. Additionally, RNA-enzyme mutations or genomic/transcriptomic RNA expression data can serve as predictive resistance biomarkers, enabling individualised therapeutic plans.

## CONCLUSION

Epitranscriptomics presents a potentially valuable approach for therapeutic interventions, leveraging the manipulation of RNA modifications to influence gene expression. Modifications like m6A, m5C, and pseudouridine are critical for fundamental RNA metabolic processes, encompassing stability, translation, and degradation. Variations in these modifications have been associated with a range of diseases, including cancer, neurological disorders, and viral infections. Consequently, the targeting of these modifications could facilitate the development of innovative, precise, and disease-specific treatments, particularly for ailments that presently lack effective therapeutic options. Nevertheless, several significant challenges persist, including the precise and selective targeting of RNA modifications, the effective delivery of selective targeting to specific tissues or cell types, and the identification or circumvention of resistance mechanisms.

Additionally, our understanding of the tissue-type- and context-specific impacts of RNA modifications remains inadequate. There are still questions about how the modifications talk to each other, how they work differently when a patient is in different disease states, and how to best use RNA modifications in treatments without messing up the normal function of the cell. The next steps in epitranscriptome drug discovery will probably focus on creating RNA-modifying drugs that only affect certain types of RNA, new ways to deliver drugs like RNA-targeted nanoparticles, and ways to target multiple points in RNA regulatory pathways at the same time. Regulating non-coding RNA through epitranscriptomic control constitutes a relatively uncharted area, yet it holds considerable promise for therapeutic innovation. Despite the field's relative infancy, rapid advances in RNA biology, high-throughput screening methodologies, computational modelling techniques, and drug delivery systems underscore the importance of RNA modifications, thereby suggesting their relevance to the evolving landscape of personalised medicine. While numerous avenues for epitranscriptomic therapy exist, maximising their potential necessitates addressing existing knowledge gaps and surmounting technological constraints. Consequently, this approach is crucial for translating the potential of epitranscriptomic research into practical solutions.

## Figures and Tables

**Fig. (1):**
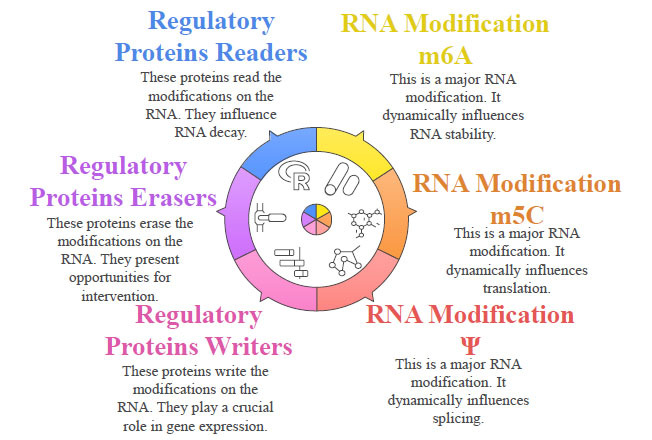
RNA molecules undergo m6A, m5C, and Ψ modifications. Writers add chemical groups to RNA and install modifications; erasers remove them and reverse modifications; readers recognise and bind to modified RNA, influencing processes such as RNA stability, protein translation, splicing, and degradation. For example, m6A modification can mark RNA for degradation, while m5C modification can enhance RNA stability. By controlling gene expression through these effects, RNA modifications support the development of therapies targeting specific gene functions in disease.

**Fig. (2):**
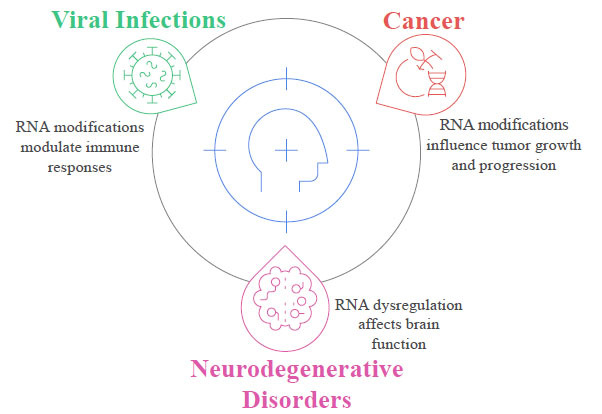
RNA modifications extensively affect the progression and development of cancer, neurodegenerative disorders, and viral infections. This growing attention has led researchers to recognise RNA modifications as potential therapeutic targets for treating multiple diseases.

**Table 1:** Summarizes various RNA modifications, their enzymatic regulation, and their functional impacts on RNA stability, translation, and cellular processes, with references to relevant studies.

**S. No.**	**RNA Modification**	**Enzymes Involved**	**Functions in Gene Expression Regulation**	**Epitranscriptomic Regulation of Cellular Processes**	**References**
1	m^6^A (N6-methyladenosine)	Writers: METTL3, METTL14; Erasers: FTO, ALKBH5; Readers: YTHDF1, YTHDC1	Regulates RNA stability, splicing, translation, and decay	Modulates differentiation, pluripotency, stress response, and apoptosis	[27]
2	m^5^C (5-methylcytosine)	Writers: DNMT2, NSUN family; Erasers: TET family (indirect); Readers: MeCP2, MBD proteins	Affects RNA stability and translation	Involved in stem cell differentiation and immune development	[28]
3	Ψ (Pseudouridine)	Writers: PUS family	Enhances RNA stability, reduces immune sensing	Facilitates stress adaptation and viral immune evasion	[29]
4	ac^4^C (N4-acetylcytidine)	Writer: NAT10	Increases mRNA stability and translation fidelity	Implicated in tumorigenesis, especially in osteosarcoma	[30]
5	m^1^A (N1-methyladenosine)	Writers: TRMT6/TRMT61A, TRMT10C	Promotes translation initiation	Regulates mitochondrial RNA translation; linked to liver and GI cancers	[31]
6	m^7^G (7-methylguanosine)	Writers: METTL1, WDR4	Supports mRNA stability, export, and ribosome binding	Key for neurodevelopment and oncogenic mRNA processing	[32]
7	m^6^Am (N6,2′-O-dimethyladenosine)	Writers: METTL4, PCIF1	Enhances mRNA stability and translation efficiency	Responds to hypoxia and controls cell cycle gene expression	[33]

**Table 2:** RNA modifications in human diseases.

S. No.	**Disease**	**Dysregulated Modification**	**Affected Gene/Pathway**	**Therapeutic Target**	**References**
1	AML (leukemia)	m^6^A hypermethylation	MYC, METTL3	METTL3 inhibitors (STM2457)	[64]
2	Glioblastoma	FTO overexpression	PD-1, MYC	FTO inhibitors (FB23-2)	[65]
3	Alzheimer’s disease	A-to-I editing loss	GRIA2, BACE1	ADAR1 activators	[66]
4	COVID-19	Viral m^6^A mimicry	IFN pathway	METTL3 blockade	[67]
5	Parkinson’s disease	m^6^A hypomethylation	SNCA (α-synuclein)	METTL3 activators	[68]
6	Breast cancer	m^6^A reader YTHDF1 upregulation	EGFR, HER2	YTHDF1-targeting PROTACs	[69]
7	Hepatitis B/C	m^6^A in viral RNA	HBV/HCV replication	FTO inhibitors	[70]
8	Autism spectrum disorder	m^5^C dysregulation	SHANK3, MECP2	NSUN2 modulators	[71]
9	Osteosarcoma	ac^4^C upregulation	Cell cycle and translation machinery	NAT10 inhibitors	[72]
10	Liver cancer, GI tumors	m^1^A dysregulation	Mitochondrial ribosomal genes	TRMT10C modulators	[73]
11	Brain tumors, developmental delay	m^7^G overexpression	eIF4E, mRNA capping genes	METTL1/WDR4 inhibitors	[74]
12	Hypoxia-related disorders	m^6^Am imbalance	Hypoxia-inducible genes	PCIF1-targeting molecules	[75]

## LIST OF ABBREVIATIONS

AD: Alzheimer’s Disease
ALS: Amyotrophic Lateral Sclerosis
AML: Acute Myeloid Leukaemia
ASOs: Antisense Oligonucleotides
CNNs: Convolutional Neural Networks
CVD: Cardiovascular Disease
lncRNAs: long non-coding RNAs
LNP: Lipid Nanoparticle
miRNAs: microRNAs
mRNA: messenger RNA
ncRNAs: non-coding RNAs
NSCLC: Non-small Cell Lung Cancer
PD: Parkinson’s Disease
RBPs: RNA-binding Proteins
RNNs: Recurrent Neural Networks
rRNA: Ribosomal RNA
siRNAs: Small Interfering RNAs
